# Editorial: Regulatory T Cells

**DOI:** 10.3389/fcell.2022.898132

**Published:** 2022-04-21

**Authors:** Fadi Issa, Jian Gu, Ling Lu

**Affiliations:** ^1^ Nuffield Department of Surgical Sciences, University of Oxford, Oxford, United Kingdom; ^2^ Liver Transplantation Center, First Affiliated Hospital of Nanjing Medical University, Nanjing, China

**Keywords:** regulatory T cells, transplantation, immunotherapy, cell biology, immune balance

Regulatory T cells (Tregs) are a subset of CD4^+^ T cells that maintain tolerance to self-antigens, prevent autoimmune disease, and limit anti-tumor immunity. Over recent years, Tregs have demonstrated great potential as either a target or immunotherapy in a variety of diseases, such as autoimmunity, cancer, transplantation and graft-versus-host disease (GVHD). In clinical trials, Tregs have been infused as an adoptive cellular therapy in diabetes, GVHD and organ transplantation. For example, in Nanjing Medical University in China, Dr. Ling Lu and his team have infused exvivo expanded nTregs to liver transplant recipients in combination with a reduction in immunosuppression to promote immune tolerance. As of writing, 5 patients out of 17 have ceased immunosuppression with the majority of the remaining patients receiving a reduced dose. Some other trials are also ongoing, both in GVHD and transplantation, including the Phase IIb TWO Study in Oxford. However, some challenges must be overcome before the implementation and widespread use of Treg therapy. This Research Topic describes the latest advances in the basic science and clinical application of Tregs, including insights into the use of Tregs for the treatment of tumors and autoimmune diseases, and new strategies for Treg therapy in organ transplantation.

The phenotypic and functional diversity of Tregs has important implications for their biology and function. Tregs can mirror conventional T cell subsets, and include naïve, effector, and memory subpopulations. Here, Sjaastad et al. from the University of Minnesota focus on the ontogeny, phenotype and function of different effector Treg subsets, which is important for the future development of tools for specific subgroups and their functions.

Tissue Tregs, also known as tissue-resident Tregs, do not recirculate in the blood or lymphatics and are adapted to the specific tissue environment. In this Research Topic Shao et al. review the phenotype, function, and cytokine expression of these Tissue Tregs. In local tissues, Tregs can restrain immune responses, maintain tissue homeostasis, and promote tissue recovery. With the increasing number of chronic tissue inflammatory diseases and immune deficiency diseases, understanding the role that Tregs play in either the control or perpetuation of these pathologies is crucial.

Current studies suggest that Tregs may be a potential treatment for ischemia-reperfusion injury (IRI). Butyric acid (BA) is a product formed by intestinal microorganisms after decomposing indigestible food and is a potential negative immunomodulator. Chen et al. show that the heme oxygenase 1(HO-1)/STAT3 signaling pathway is related to the inhibitory effect of BA on the differentiation of Th17 cells. BA regulates the differentiation of Th17 cells into Tregs and reduces renal IRI. This provides a route for BA to inhibit inflammation by regulating the balance of Tregs to Th17 cells.

Human amniotic mesenchymal stem cells (hAMSCs) have the potential for multi-directional differentiation and the ability to promote immune regulation and tissue repair in a number of diseases. Heren, Deng et al. provide convincing evidence for the hAMSC and Treg combined activity. The authors find that Tregs improve the function of hAMSCs by regulating the TGF-β/IDO signaling pathway, increasing cytokine expression of hAMSCs, and enhancing the effect of liver cirrhosis treatment. This suggests that co-infusion of hAMSCs and Tregs may provide a promising method for the treatment of liver cirrhosis.

Tregs can impair anti-tumor responses and reduce the efficacy of immunotherapies. In the past few years, there has been interest in targeting Tregs to enhance anti-tumor responses. As TNFα plays a key role in phenotypic stabilization and inhibition of human and mouse Tregs, tumor necrosis factor receptor 2 (TNFR2) has recently been identified as a target for anti-cancer immune checkpoint therapy Moatti and Cohen have reviewed and analyzed the results of recent studies on TNFR2 function in Tregs. This signaling pathway may provide a new route for anti-tumor immunotherapy.

Altering the balance of effector and regulatory cells is likely to be the key to the control of alloimmune responses in transplantation. While the most focus has been on T cell control, B cell activity must also be controlled to ensure that late alloresponses and chronic allograft dysfunction are also targeted. Attention is therefore also turning to regulatory B cell subpopulations or cells that control B cells in the lymphatics. In the accompanying article, Chong et al. summarize new discoveries in T follicular regulatory cells, regulatory B cells, and alloreactive tolerogenic B cells and their roles in the control of allo-immunity and transplant rejection.

Non-human primates (NHP) are valuable for the translation of Tregs from basic research to clinical application. Thomson and Thomson et al. have contributed crucial data over the years to assist in the translation of Treg therapeutics to the clinic, with a particular focus on alloreactive Tregs. In this Research Topic, the authors explore the approaches used to produce NHP Tregs as well as the differences between polyclonal and alloantigen reactive Tregs. Important insights are provided in the methodology for Treg production and assessment in NHPs, as well as future perspectives on their therapeutic potential.

Adoptive transfer of *ex vivo* expanded Tregs is a key method under investigation for the treatment of immune-mediated diseases. Ou et al. describe the epigenetic changes in Tregs after repeated cell stimulation which may affect the survival and function of cells. The authors call for a careful examination of the molecular changes in the production of T-cell products, and their research also highlights the importance of epigenetic analysis in evaluating the quality of Treg cell products in the future.

Another major challenge for Treg therapy use in the clinical setting is the need for cryopreservation, which may impact Treg viability and function. Kaiser et al. examine the effects of different cryoprotectants on Tregs, finding that a medium containing 5% dimethyl sulfoxide enhances cell viability, recovery, and function after thawing. This is important data for Treg clinical translation.

Overall, the studies and overviews in this Research Topic highlight key points of progress in our understanding of Tregs and their therapeutic application. Progress has been across a broad front across all aspects of Treg biology, together with building crucial foundations for the deployment of Tregs to the clinic. Ongoing clinical trials are likely to report their results in the near future, and these results are highly anticipated.

